# Marine Delivery Vehicles: Molecular Components and Applications of Bacterial Extracellular Vesicles

**DOI:** 10.3390/md22080363

**Published:** 2024-08-09

**Authors:** Angela Casillo, Raffaele D’Amico, Rosa Lanzetta, Maria Michela Corsaro

**Affiliations:** Department of Chemical Sciences, University of Naples Federico II, Complesso Monte S. Angelo, 80126 Naples, Italy; raffaele.damico@unina.it (R.D.); lanzetta@unina.it (R.L.); corsaro@unina.it (M.M.C.)

**Keywords:** marine bacteria, outer membrane vesicles, chemical structure

## Abstract

In marine ecosystems, communication among microorganisms is crucial since the distance is significant if considered on a microbial scale. One of the ways to reduce this gap is through the production of extracellular vesicles, which can transport molecules to guarantee nutrients to the cells. Marine bacteria release extracellular vesicles (EVs), small membrane-bound structures of 40 nm to 1 µm diameter, into their surrounding environment. The vesicles contain various cellular compounds, including lipids, proteins, nucleic acids, and glycans. EVs may contribute to dissolved organic carbon, thus facilitating heterotroph growth. This review will focus on marine bacterial EVs, analyzing their structure, composition, functions, and applications.

## 1. Introduction

Extracellular vesicles (EVs) are small lipid-bilayer nanospheres (about 40 nm–1 µm diameter) secreted from cells belonging to the three domains of life [1,2,3] and vary in their morphology, biogenesis, composition, and biological role [2]. Although initially underappreciated and considered cellular debris, biological fluids can contain large quantities of EVs that shuttle various molecules from parental cells to other cells, including proteins, genetic material, and toxins. The first studies on EVs focused on Eukarya EVs, particularly those produced from mammalian cells, for their role in the struggle with infection and in the control of normal physiological and disease processes [2,3,4]. 

Starting from mammalian EVs, the production of extracellular vesicles has been recognized to be a common feature in bacteria, fungi, plants, and algae.

EVs fulfill a myriad of functions and are recognized as important vehicles of long-range intercellular communication, especially during stress conditions and host–pathogen interactions. Considering their ability to move into biological fluids, EVs are now considered promising biomarkers for disease diagnosis and therapeutic applications [5,6,7].

In marine environments, bacteria and cyanobacteria account for >90% of the total oceanic biomass [8]. Although largely unexplored, they provide a useful source of natural products with high-value biotechnological potential. During the last two decades, the scientific community has focused attention on bacterial extracellular vesicles (BEVs) [9,10,11] involved in cell-to-cell interactions [2,3,4], virulence [12], horizontal gene transfer [13], biofilm formation [14], and quorum signaling [15,16]. 

Interestingly, while for pathogenic [12,17] and gut [18,19,20] bacteria, the secretion of BEVs and their functions have been investigated, the state of the art in the marine environment is still largely uncharted. Indeed, researchers are trying to shed new light on these structures’ diverse roles in microbial ecology. One possibility is that, in addition to that found for pathogens, the functions of marine BEVs could guarantee survival in an environment where the nutrients are poor [21]. Another option is that the vesicles can carry a higher number of chemical effectors, so the cells have to produce a lower number of molecules. The final effect is less energy expenditure for the bacterial cell [22]. 

More intriguing is the recent hypothesis that EV production has a functional role since it could act as a long-distance communication pillar by exploiting the large membrane area of the vesicles [23]. All these hypotheses are valuable and show that the topic is attractive and scientifically relevant. This review will focus on marine BEVs since there is a growing interest in finding out the role of the vesicles in bacteria in the marine microbiome and how these nanoparticles can facilitate cross-feeding or cross-talking inside microbial communities in seawater columns [11,12,13,14,15,24,25,26]. Moreover, the BEVs’ motion over long distances implies that these structures are responsible for marine carbon flux and may modulate the growth of heterotrophic communities [16]. Indeed, the possibility that the marine currents impair the BEV cross-talking with other cells could be overcome by the presence of high concentrations of bacterial, virus, and cyanobacterial cells in a tiny volume of seawater, which guarantees the closeness of individual cells [27]. Finally, and no less importantly, beyond their ecological significance, vesicles produced by marine organisms hold promise for biotechnological applications. The molecular cargos encapsulated within these vesicles, including enzymes, metabolites, and genetic material, present opportunities for bioprospecting and biotechnological innovation. 

## 2. Biogenesis of Bacterial Vesicles

BEVs are produced during normal growth, and stress can influence their production. In some cases, abiotic factors, including changes in temperature, nutrient availability, reactive oxygen species, and UV exposure, correlate with increased vesicle production. The release of EVs may also be induced by intracellular stimuli, such as the accumulation of peptidoglycan (PG) fragments and LPS [28]. In *Cylindrospermopsis raciborskii* vesicle formation is accompanied by phosphatidylserine exposure, a molecular event also observed in EV-secreting eukaryotic cells [29]. 

There are several types of BEVs depending on the microorganism’s type (Gram-positive or Gram-negative) and the way in which they are generated [9].

**Figure 1:**
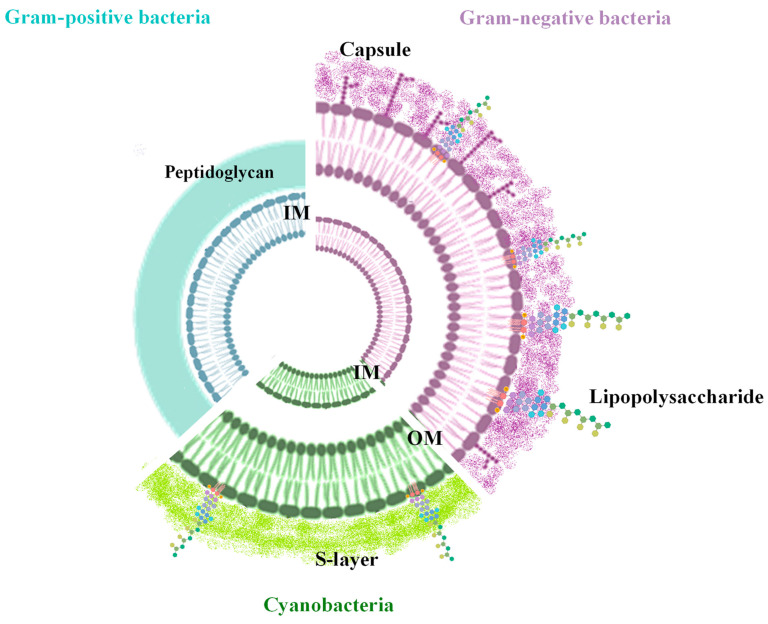
Cell wall organization of Gram-positive, Gram-negative, and cyanobacteria.

Gram-negative bacteria possess a cell wall of two phospholipid-enriched membranes spaced by a thin peptidoglycan layer (PG) (Figure 1). The outer membrane (OM) comprises proteins, phospholipids, and lipopolysaccharides (LPSs) [30], whereas the inner membrane (IM) is a fluid phospholipid bilayer. In Gram-positive cell walls, as much as 90% is a single type of molecule, the peptidoglycan, although teichoic acids are usually present in small amounts (Figure 1). Furthermore, both Gram-negative and Gram-positive cell walls can be decorated by a polysaccharide material, forming a capsule. 

**Figure 2:**
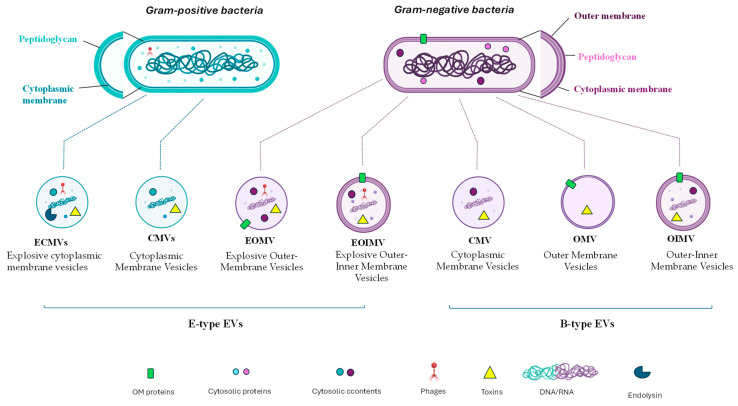
Biogenesis of bacterial extracellular vesicles. The EVs are produced through two different mechanisms: explosive cell lysis (E-type), and blebbing of the outer membrane (B-type). Gram-positive bacteria produce ECMVs and CMVs (left), while Gram-negative bacteria produce both B-type EVs (OMVs and OIMVs) and E-type EVs (EOMVs, CMVs, and EOIMVs).

So far, two ways in which Gram-negative bacteria generate vesicles have been identified: the blebbing of the outer membrane (B-type or non-lytic EVs) [31] and explosive cell lysis (E-type or lytic EVs) (Figure 2) [32]. Non-lytic biogenesis can produce different types of vesicles (Figure 2): outer membrane vesicles (OMVs) and outer inner membrane vesicles (OIMVs). The first (OMVs) consist of a single membrane bilayer (OM) and relative glycolipids and proteins associated with them and are devoid of cytoplasmic components. The OIMVs comprise two membrane bilayers derived from IM and OM, respectively, and separated from the peptidoglycan. Finally, cytoplasmic membrane vesicles (CMVs) have been discovered (Figure 2) [28]. All these vesicles are the results of stress on the cell wall, such as antibiotics or environmental conditions [9,28,33]. 

Conversely, E-type EVs are the result of cell lysis that can be mediated by the activation of prophages and enzymes or can occur during cell death. A phage-derived endolysin determines the weakening of the PG, which is followed by an explosion [9] and the self-annealing of membrane fragments. These events can generate two subtypes of vesicles (Figure 2), explosive outer-inner membrane vesicles (EOIMVs) and explosive outer-membrane vesicles (EOMVs), depending on the presence of a single or double-layered membrane [9,28,33]. 

Gram-positive bacteria have been demonstrated to form two types of explosive EVs, both containing cytosolic material (Figure 2). In both cases, the vesicles consist of a single membrane bilayer (IM) and are the result of the disturbance of the PG triggered by endolysins [9]. The derived EVs are named explosive cytoplasmic membrane vesicles (ECMVs). 

Since the loss of integrity of the cytoplasmic membrane determines the death of the cells, this mechanism is also called “bubbling cell death” [9]. Conversely, when the EV formation is due to an exogenous endolysin or antibiotics, the produced EVs do not contain endolysin CMVs [9,34,35]. 

Finally, although the production of EVs on the cell walls of cyanobacterial strains has been demonstrated by microscopy images, the detailed mechanism regarding EVs’ biogenesis in these microorganisms is still very limited [11,13]. 

## 3. Size and Molecular Components of the BEVs

**Figure 3:**
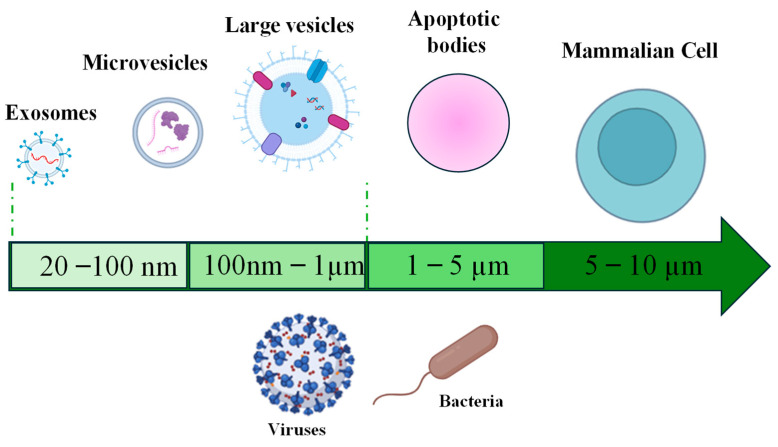
Dimensions of vesicles produced from both Eukarya and Bacteria.

Vesicles produced by eukaryotic cells are classified as exosomes (30–100 nm) originating from the endosomes, microvesicles (100 nm–1 μm) released from the cell membrane, and apoptotic bodies (1–5 μm), which are residual cells that have undergone apoptosis or programmed cell death (Figure 3) [9]. In mammalian cells, exosomes are involved in immune modulation and disease progression, as observed for vesicles released by breast cancer cells that contribute to metastasis. Microvesicles in human cells are important for cell communication and pathology, as exemplified by vesicles from platelets, which aid in blood clotting and inflammation. Large vesicles, like apoptotic bodies, have been found in various eukaryotic cells, such as those from *Saccharomyces cerevisiae*, involved in removing cellular waste and signaling during cell death [36]. 

Marine bacteria and cyanobacteria show the tendency to produce a higher number of larger vesicles compared to many eukaryotic cells, exhibit a diverse range of sizes, and are classified into small vesicles (30–100 nm), medium-sized vesicles (100–300 nm), and large vesicles (300–700 nm) (Table 1). Small vesicles, also named exosomes, as those produced by *Prochlorococcus* cyanobacteria, are involved in intercellular communication and can carry proteins, lipids, and nucleic acids. Medium-sized vesicles, also classified as microvesicles or ectosomes, play roles in nutrient acquisition and environmental signaling, as represented by the cyanobacteria *Synechococcus*. Furthermore, the large vesicles, like those released by *Vibrio cholerae*, are involved in cellular waste removal and can contain fragmented DNA and other cellular debris [37]. Finally, *Shewanella* species release large vesicles (up to 700 nm) that contain extracellular polymeric substances and metabolic bioproducts, highlighting their role in biofilm formation and environmental adaptation [38]. These examples illustrate the diversity in vesicle sizes and functions across different organisms, emphasizing how size influences their biological roles and interactions within their respective environments. 

Following the most recent papers about the structure of membrane vesicles, the components specified below are described.

### 3.1. Proteins

Proteins in vesicles have been demonstrated to be cytoplasmic, periplasmic, inner and outer membrane proteins (OMP) [42,43], virulence factors [44], enzymes, and proteins involved in biofilm formation [45]. *Alteromonas macleodii* KS62 has been reported to produce OMVs, the protein content of which is very rich in hydrolytic enzymes (30% of the proteome) [46]. This is not surprising since the hydrolytic enzymes are necessary for nutrient supply and colonization surfaces. Similarly, for *Bacteroides fragilis*, a gut microbiota bacterium, it has been suggested that the EVs equipped with hydrolytic enzymes could facilitate the recruitment of the necessary nutrients for the entire microbiota bacterial community [47].

The production of OMVs containing hydrolytic enzymes was also found in a pool of *Alteromonas macleodii* strains [48]. For all the examined strains, despite the presence of two different populations of OMVs due to their different sizes, the content of hydrolytic enzymes was high. In addition, the presence of proteins probably involved in bacterial adhesion processes was observed.

Many BEVs are composed not only of membrane proteins but also cytoplasmic and periplasmic ones. This is not true for the marine extremophile *Novosphingobium pentaromativorans*, for which the proteomic analysis of the vesicles indicated most exclusively the presence of OMPs [49]. The authors suggested that for this bacterium, the possibility of loading cytoplasmic cargo proteins in the vesicles could be hampered by the high salts and low nutrients available in its natural environments. A family of marine Gram-negative bacteria of particular interest is *Vibrio*. This comprises both pathogens and non-pathogenic bacteria. Among the former, *Vibrio cholerae* has been found to produce a higher number of vesicles after shifting from the aquatic environment to the infected host [49]. It is possible that the vesiculation is augmented to eliminate unfavorable compounds from the outer membrane and better colonize the host environment. The bacterium regulates protein expression (and the lipid A structure, see below) to adapt to different environments. The expression of porin OmpT in place of OmpU in Vibrio cholerae affects the pathogenesis mechanism and promotes resistance to bile and the ability to colonize [50]. In another paper, *Vibrio cholerae* cells and vesicle proteomics have been compared [51]. The study demonstrated that the vesicles were enriched in virulence factors concerning the cells. The authors hypothesize that this enrichment points to the theory that the vesicles are not simply the product of membrane blebbing but a programmed way to transport vehicle molecules [51].

Noteworthy is the finding that the coral-pathogen *Vibrio shilonii* can deliver BEVs to send signals to the holobiont animal [16]. The vesicles were found to contain ectohydrolases, which are crucial molecules for marine bacteria since they are directly involved in the carbon cycle [27]. Li et al., through a proteomic approach, identified double-layered vesicles since both cytoplasmic and outer membrane proteins were found. The hypothesis is that the cargo composition can be useful in stimulating the immune system of the coral. Moreover, the same authors found that the production of BEVs can be associated with a defense mechanism against phages and coral pathogens [52].

The protein profile of the OMVs from *Pseudomonas syringae* Lz4W, an Antarctic isolate, comprises OMPs, lipoproteins, ABC transporters, ribosomal proteins, cytosolic enzymes, and many others [53]. Kulkarni and co-authors underlined that OMVs from *P. syringae* Lz4W are involved in antibiotic resistance and sensitivity. The mechanisms of action played by the vesicles seem to depend on the environmental situation. In addition, since phospholipids and LPSs from cold-adapted bacteria are different from corresponding mesophiles due to the higher amount of unsaturated fatty acids necessary for membrane fluidity at low temperatures, the packing parameters of the membrane are different for this bacterium.

The genus *Shewanella* is prone to the production of OMVs, as revealed by the species *livingstonensis* AC10 [54], *vesiculosa* M7 [55,56], and HM13 [57]. All these strains are cold-adapted with a putative consideration for the secretory production of proteins in the extracellular environment. Proteomic studies have been performed for *S. vesiculosa* M7 vesicles, revealing that this bacterium can produce a new type of vesicle named outer-inner membrane vesicles (EOIMVs; see Figure 1). The last possesses a double-bilayered structure harboring cytoplasmic and plasma membrane proteins and can incorporate DNA [56]. Unlike the M7 strain, the vesicles of HM13 have been carefully characterized for the presence of a cargo protein named P49, whose function is still unknown [57]. Interestingly, *S. vesiculosa* HM13 also produces a putative sensor protein involved in the suppression of biofilm formation [58].

### 3.2. Nucleic Acids

Nucleic acids associated with vesicles play significant roles in marine microbial communities and ecosystem dynamics [59]. The incorporation of nucleic acids into vesicles in marine bacteria and cyanobacteria takes place through several mechanisms. One mechanism involves the passive encapsulation of nucleic acids within the vesicle lumen. For example, *Prochlorococcus* releases vesicles that contain a mix of RNA and DNA, which are captured as the vesicle buds off of the cell membrane. This process does not require specific targeting mechanisms and is influenced mainly by the physical dynamics of vesicle formation [38].

Marine bacteria and cyanobacteria utilize molecular chaperones and RNA-binding proteins to recognize and sort nucleic acids into vesicles. These proteins can bind specifically to nucleic acids and facilitate their incorporation into the forming vesicles, ensuring the selective packaging of functional genetic material. For instance, *Vibrio cholerae* employs molecular chaperones such as Hsp70 (heat shock protein 70) and RNA-binding proteins like Hfq. These proteins recognize and bind to specific RNA sequences, facilitating the packaging of regulatory RNAs into vesicles [40].

Furthermore, in *Synechococcus*, membrane-associated complexes, such as the ExoU complex, which play a crucial role in sorting and packaging nucleic acids into vesicles, have been reported. These complexes facilitate the selective incorporation of DNA fragments into vesicles, including those encoding genes related to photosynthesis and nitrogen fixation [60].

Finally, the interaction between marine bacteria, cyanobacteria, and viruses (phages) influences nucleic acid incorporation into vesicles. For example, cyanophages infecting *Prochlorococcus* cyanobacteria have been shown to package their DNA into vesicles released by infected cells, leading to the co-presence of host and viral genetic material in the environment, enhancing the potential for horizontal gene transfer [13].

The incorporation of nucleic acids into vesicles significantly enriches their functional repertoire, contributing to various biological processes in marine microbial communities: serve as vectors for horizontal gene transfer (HGT), facilitating the dissemination of genetic material, including antibiotic resistance genes, metabolic pathways, and virulence factors, among microbial populations [38,59]. For instance, vesicles released by *Vibrio cholerae*, *Pseudomonas aeruginosa*, *Synechococcus*, and *Shewanella* genera contain both functional genes, facilitating HGT in marine environments and DNA-encoding bacteriocins that inhibit the growth of competing bacterial species [40,60]. Nucleic acids may encode regulatory elements, such as small regulatory RNAs (sRNAs), microRNAs (miRNAs), and transcription factors, which modulate gene expression and cellular responses to environmental cues [29,61]. Vesicles released by cyanobacteria belonging to the *Synechococcus* genus contain miRNAs involved in regulating photosynthesis and nitrogen metabolism in recipient cells [61]. Vesicles released by *Vibrio parahaemolyticus* and *Vibrio cholerae* carry DNA fragments encoding virulence genes, enhancing the pathogenic potential of these bacteria [52,60]. *Shewanella* spp. vesicles contain DNA fragments encoding chemotaxis proteins involved in sensing environmental gradients [38].

### 3.3. Phospholipids

Phospholipids in vesicles play a fundamental role in cargo selection and transport. Even if some sphingolipids have been demonstrated to be delivered for a long distance through vesicles in Bacteroides species [62] the importance of characterization of the lipid fraction in BEVs has been overlooked in many papers. Essential parts of the biogenesis of membrane vesicles are the structures of the fatty acids. The last ones are usually involved in maintaining the fluidity or rigidity of the membrane, which is particularly important for microorganisms thriving in cold environments. A few papers describing the phospholipid structure of bacterial membrane vesicles are devoted to studying cold-adapted bacteria. Antarctic *Pseudomonas syringae* has been described as a producer of vesicles containing phospholipids with both saturated and unsaturated fatty acids [53]. This was expected since the increase in membrane fluidity of cold-adapted bacteria necessary to survive at low T entails the biosynthesis of unsaturated fatty acids [63]. In the case of another cold-adapted bacterium, *Pseudoalteromonas antarctica*, only phosphatidylethanolamine and phosphatidylglycerol have been reported [64].

An enhancement in the production of membrane vesicles has been observed for a change in phospholipid biogenesis with another Gram-negative bacterium named *Shewanella livingstonensis* Ac10. A depletion of the gene for the biosynthesis of eicosapentaenoic acid (EPA) induced a significant and quantitative increase in vesicle production [55]. It was suggested that the lack of EPA fatty acid could alter the protein composition of the vesicles since, in these conditions, the transfer of a misfolded OmpC176 was facilitated.

Some marine bacteria can alter the molecular surface in response to different environments. The key case is represented by *Vibrio cholerae,* for which a change in the phospholipid composition moving from the marine to the host environment was observed. Zingl et al. [65] reported that phospholipid accumulation on the membrane surface can be related to membrane vesicle release. After *Vibrio* enters host cells, it has been observed that there is a change in the lipid moiety of the LPS (see below), a consequent change in the asymmetry of the outer membrane, and an accumulation of phospholipids [34]. The different ratios of phospholipids/LPS are crucial to producing the vesicles. Among the factors regulating the increase in BEV production is the repression of the VacJ/Yrb transporter, influenced by the depletion of iron [34] and sulfur [66].

*Vibrio* species can produce CAI-1, a long-chain amino ketone, a signal molecule involved in so-called quorum sensing, a way of communication among microorganism cells [67,68]. In some cases, the QS molecules can also be associated with vesicles, as has been reported for *Vibrio harveji* strain MR17. The loading of this molecule is probably due to its lipophilic character, which allows its interaction with the phospholipid bilayer and LPS, facilitating its distribution among bacterial cells [68].

### 3.4. Lipopolysaccharides

Lipopolysaccharides are the main components of the outer membrane of Gram-negative bacteria, of which they constitute 75% of the outer leaflet [69]. LPS is one of the most well-studied pathogen-associated molecular patterns (PAMPs) since it is a powerful activator of innate immune responses [70,71]. LPS binds to the proteins Toll-like receptor 4 (TLR4) and myeloid differentiation factor-2 (MD2) to activate pro-inflammatory signaling pathways. The TLR4–MD2 receptor complex is crucial for the host’s recognition of Gram-negative bacterial infection [72]. These molecules are composed of three different domains: lipid A, embedded within the outer leaflet of the outer cell membrane, an oligosaccharide named “core”, and a polysaccharide mentioned as O-antigen that sticks out in the extracellular environment [73,74,75,76]. Since the biogenesis of vesicles in Gram-negative bacteria is generated directly from the outer membrane, the lipopolysaccharides are particularly abundant in EVs. Nevertheless, their structures and the roles they eventually played in transportation have been only barely understood.

In some pathogenic Gram-negative bacteria, it has been demonstrated that the structure of the LPS components is involved in the vesiculation process. *P. aeruginosa* is reported to produce two different O-chains, namely A and B-bands, respectively. The A-band is a hydrophobic D-rhamnan chain, whereas the B-band displays negative charges due to the presence of acidic monosaccharides. The repulsion among the polysaccharide chains of the B-band could be responsible for a different curvature of the outer membrane, thus releasing a higher number of vesicles [12]. Differently from *Pseudomonas aeruginosa*, *Salmonella enterica* serovar Typhimurium is involved in a novel mechanism for OMV biogenesis where the lipid A modification is involved in a remodeling event caused by the induction of the PagL enzyme [77]. Feldman’s group has clarified that the LPS can play a role in BEV biogenesis [78]. They proposed the presence of a peculiar cargo selection process in which the lack of some fatty acids on the lipid A moiety isolated from the *Porphyromonas gingivalis* BEVs is responsible for the insertion of different proteins on the vesicles. Instead, for the same bacterium, they demonstrated that there is no involvement of the O-chain in vesicle formation. Differently from *P. gingivalis*, a study performed by the same research group on *Bacteroides fragilis*, showed that there were no differences in the lipid A structures between cells and vesicles [48].

Very few structures of LPS from marine EVs have been isolated and characterized. The molecular characterization of the LPS from both cells and EVs of *Shewanella vesiculosa* HM13 has revealed the same structures [79,80]. The bacterium, classified as cold-adapted and isolated from the intestine of a fish, can produce abundant EVs carrying an unknown cargo protein named P49 [57]. Even though many other *Shewanella* strains have been reported to produce EVs [81], no experiments to detect the chemical structures of the LPS from these strains have been performed [55,56]. Frias et al. observed a different amount of EVs for the marine *S. livingstonensis* NF22T when the microorganism was grown at different temperatures. The lowering of temperature determines a higher amount of the recovered EVs [81]. Other marine bacteria have been studied for the content of LPS in their produced EVs, such as *Pseudoalteromonas antarctica* NF3, for which the LPS polymers from the cells and the vesicles have the same mobility on the SDS-PAGE [64]. This bacterium was particularly interesting due to the presence of an additional band for both LPS samples near the top of the gel, most probably due to a capsular polysaccharide. The negative-stained TEM images of the vesicles and the observed fibrous fringe around the cells suggested the production of extracellular polysaccharides, thus confirming the above hypothesis.

The lipopolysaccharide from cells and OMVs of *Cellulophaga lytica*, a marine Gram-negative bacterium, is involved in the process of metamorphosis for the marine worm *Hydroides elegans* [82]. A bioassay-guided fractionation of the molecular components of OMVs from *C. lytica* indicated that LPS was responsible for the larval settlements. The authors hypothesized that the induction of larval settlement and metamorphosis is strain-specific due to the inherent structural variability of LPS.

### 3.5. Capsular Polysaccharide

Among the bacterial surface glycans, capsular polymers occupy an escalating position due to their involvement in many biological processes, such as engagement with biofilm formation [83], pathogenesis mechanisms [84], nutrients, and involvement in the biogeochemical cycling of elements in the oceans [85]. Polysaccharides contribute to the formation of the extracellular polymeric substances (EPS) biofilm matrix, in which bacterial vesicles are entrapped [41].

Capsular polysaccharides are strictly associated with the outer membrane of both Gram-positive and Gram-negative bacteria [86], the presence of which can be revealed by microscopy. The polysaccharide can be retained on the surface by a lipid moiety [87] or by ionic interactions [88], since most of these polymers are anionic. Bacteria can also produce exopolysaccharides, which are secreted into the surrounding environment [89].

Since the generation of BEVs occurs through mechanisms involving the outer membrane, it is reasonable to find a layer of capsular polysaccharide around the vesicles.

Capsular polysaccharides from pathogenic bacteria are classified as PAMPs, and therefore they are among the preferred subjects for the construction of vaccines. *E.coli* OMVs, used as a platform to deliver capsular polysaccharides against *Streptococcus pneumoniae*, were found to induce a significant immune response [90]. Also, engineered *E.coli* were able to produce recombinant vesicles carrying the capsular PNAG, which induced the formation of IgG antibodies after immunization in mice [91].

Marine bacteria capable of producing cells covered by capsular polysaccharides have been reported for *Shewanella* strains, and the presence of such polymers together with smooth LPS has been related to the surface-strong adhesion capacity of members of this genus [92]. In a paper from Mercade’s group, it has been reported that growths of cold-adapted bacteria belonging to various genera of class *Gamma proteobacteria* revealed the presence of a large amount of extracellular material together with BEVs [81]. It was speculated that the reasons for which extracellular matter was abundant could be to constitute a microenvironment for the survival of bacterial cells. In addition, when the temperature is low, it has been demonstrated that capsular polysaccharides can play a cryoprotectant role [93,94], whereas exopolysaccharides can protect from desiccation, enhance metal chelation, scavenge nutrients and small molecular compounds from solution, and aid cell motility and adherence [95]. Recently, a capsular polysaccharide isolated from both the cells and EVs of *Shewanella vesiculosa* HM13 has been characterized [96]. It is composed of a pentasaccharide repeating unit containing three aminosugars, of which one is a new monosaccharide named shewanosamine. The structure of this capsule is peculiar since it is characterized by a subtle equilibrium between hydrophilic and hydrophobic features. In the study, Casillo et al. [96] observed the formation of a “polysaccharide corona” on the surface of both synthetic polystyrene and liposome nanoparticles, thus demonstrating the strong adhesive properties of this polysaccharide. Capsular polysaccharides from some other cold-adapted and marine bacteria have been characterized [93,94,97,98,99]. Intriguingly, all of them show the presence of aminosugars, hydrophobic moieties, and ionic groups. We could speculate that these features are necessary for adhesion on biotic and abiotic surfaces. In addition, the sticky behavior of these molecules is certainly exploited for biofilm formation. It has been reported that the MVs have a pivotal role in starting biofilm formation [100,101], and then the capsular polysaccharide may take an active part in this event.

Changes in the environment can create stress for microorganisms [102], and bacterial vesicles and their components are involved in both production amounts and functional differences. However, a distinct role due to the presence of capsular polysaccharides on the MVs is far from being clarified.

## 4. Conventional Techniques for BEV Visualization, Purification, and Characterization

The isolation and purification of BEVs is a difficult task due to the possibility of recovering the vesicles together with non-EV materials, such as flagella, pili, phages, protein complexes, and DNA-protein complexes. For these reasons, shared protocols have been set up and published by the International Society for Extracellular Vesicles (2014, 2018, and 2024) [103]. The protocols have been regularly updated since 2014 and report both separation and characterization methods [104].

**Figure 4:**
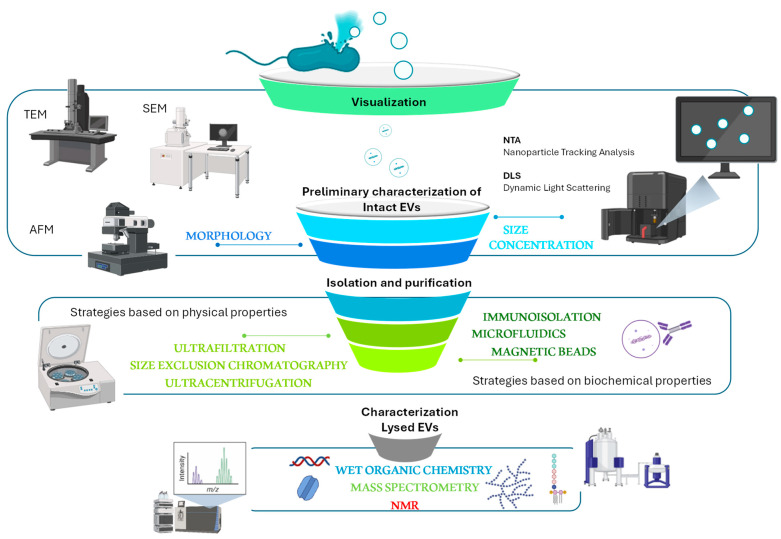
Main purification and characterization of BEVs methods. EVs are preliminary characterized through microscopic analyses to obtain morphological information and by using DLS and NTA for determining the concentration and size distribution of particles. To obtain details about the chemical composition, the purified EVs are subjected to chemical analyses.

To be sure that a bacterium produces membrane vesicles, methods for their visualization are necessary (Figure 4). The large majority of marine BEVs have been visualized by negative-stained TEM (Transmission Electron Microscopy) [46,49,57,64,81,82,105,106,107,108,109,110] SEM (Scanning Electron Microscopy), epifluorescence microscopy, and Atomic Force Microscopy (AFM). FE-SEM (Field Emission-Scanning Electron Microscopy) analysis has been used for *Shevanella vesiculosa* HM13 to observe the surface morphology of the cells secreting BEVs, thus demonstrating the absence of cell lysis while producing vesicles [57]. Finally, super-resolution microscopy (Cryo-EM) of cryo-electron micrographs allowed the visualization of a large periplasm, a protrusion of the cytoplasm, and tubular appendages [109].

The characterization of BEVs is carried out by taking into account the physical state of the sample, both native vesicles and lysed vesicles, by considering their shape, size distribution, concentration, surface, or internal contents (Figure 4). The preliminary optical analytical approaches that can be used are usually related to the physical state of the isolated vesicles. The intact EVs can be analyzed in the form of a dynamic suspension by using NTA, which gives information about particle number and size distribution [111,112], dynamic light scattering [113], but also fluorescence correlation spectroscopy (FCS) and high-resolution flow cytometry [11], flow cytometry, fluorescence anisotropy, live microscopy, or captured on a surface employing immunomagnetic beads, arrays, microfluidics, and microscopy on fixed samples. Instead, the analyses of lysed EVs generally require molecular analyses [109,114]. Generally, because none of these approaches can yield comprehensive data about MVs, a panel of these approaches is usually used [115].

The main activity for the purification of BEVs is reported to be differential ultracentrifugation (UC) to separate intact vesicles from cells and undesirable debris. The UC is based on solute sedimentation, according to their density and size. Usually, the UC step results in a fraction enriched by EVs but contaminated by flagella, pili, and proteins. The subsequent density gradient centrifugation is a useful step to remove contaminating protein aggregates and other cellular structures [114,116,117,118]. This method is based on the separation of EVs according to their physical characteristics, such as size, shape, and density. Gel filtration chromatography allows for the purification of the EVs from contaminants by taking advantage of the different molecular weights. Finally, precipitation and immunoisolation are also performed [114,116,117,118]. Preliminary information about BEV composition can be obtained through the quantification of suitable markers, such as proteins and phospholipids. The protein concentration can be measured by classical colorimetric methods such as Lowry or Bradford, together with stained gel-electrophoresis analysis showing at least membrane or outer membrane proteins [111,116] whereas the lipids can be measured through a fluorescent probe with a fluorometer [116].

The purification of vesicles is mandatory for the consecutive proteomic [119,120], lipid [121], nucleic acid [122], and carbohydrate analyses [123,124].

## 5. Functional Significance and Biotechnology Applications of Vesicles

Extracellular vesicles are implicated in various biological processes. One of the most recognized functions of BEVs is transmitting information between bacterial and eukaryotic cells. Furthermore, material transported through BEVs rather than excretion directly into the environment may be advantageous for the bacterium. Indeed, BEVs are more suitable for delivering microbial molecules at higher distances than a surface secretion system [125]. The human microbiota *Bacteroides fragilis* has been found to deliver to the host immune system an inflammatory molecule such as the PSA polysaccharide, where the transportation takes advantage of the vesicle system [126]. Vesicles are also involved in cell signaling since they contain quorum-sensing molecules, secondary messengers, and other signaling compounds, thus facilitating cell–cell and cell-environment communication. Vesicles can serve as vehicles for transporting nutrients such as carbon, nitrogen, and phosphorus. Indeed, in nutrient-limited environments, this mechanism allows cyanobacterial populations to efficiently scavenge and share scarce resources, enhancing their collective fitness and resilience [127].

Extracellular vesicles may carry antimicrobial compounds, toxins, and defensive proteins that help protect cells from predation, competition, and environmental stressors. Additionally, they can serve as vehicles for horizontal gene transfer, facilitating the exchange of genetic information between cells and potentially contributing to the evolution and diversification of microbial populations. Finally, they are involved in environmental interactions.

Studies have shown that *Prochlorococcus* EVs can interact with diverse microbial cells, suggesting a potential role in mediating microbial interactions and ecosystem dynamics in marine environments [13,128]. They can be taken up by other microbial cells, including bacteria, archaea, and eukaryotes, influencing their physiology, metabolism, and behavior. Additionally, vesicles released by cyanobacteria can impact the structure and function of microbial communities, shaping ecosystem dynamics and biogeochemical cycling in marine environments.

While the role of vesicles in bacterial pathogens is actively studied, the role of vesicles in the marine environment is poorly understood. Membrane vesicles were previously observed in the cyanobiont that colonizes the sporocarp of the water fern *Azolla microphylla* [129]. The authors hypothesized that vesicles could deliver soluble sugars and material for biofilm development.

**Figure 5:**
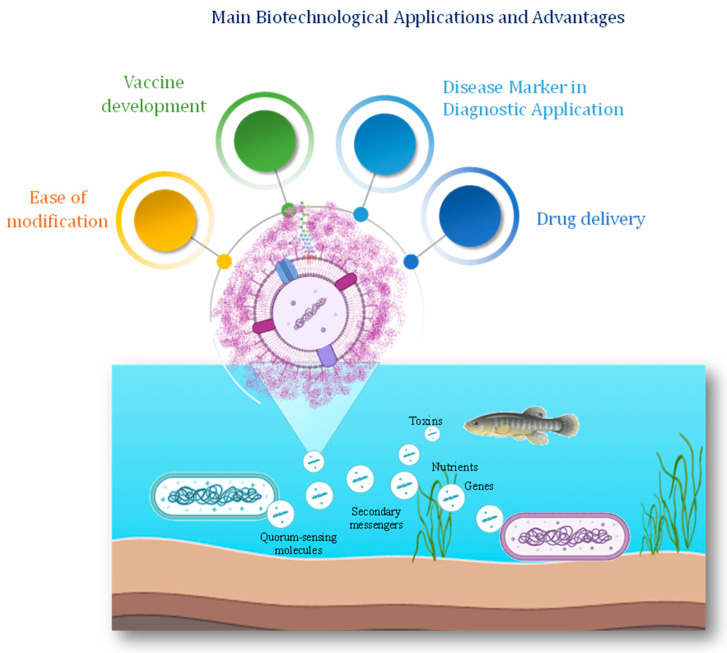
Roles of BEVs in marine ecosystems and their possible applications. Marine BEVs have been involved in cell–cell communication, including host–virus interactions, gene and toxins transfer, nutrient transport, and biofilm formation. The natural ability to deliver cargo molecules makes the BEVs a useful drug delivery system.

Recently, the applications of vesicles from marine bacteria have increased in various fields as they offer a great diversity of cargo molecules (Figure 5 and Table 2). Vesicle-associated nucleic acids hold significant biotechnological potential for various applications, including environmental monitoring, bioremediation, and biopharmaceutical production [129]. The utilization of vesicle-derived DNA for metagenomic analysis has emerged as a powerful tool for studying microbial diversity and functional potential in marine ecosystems [13].

The use of nanoparticles (NPs) for drug delivery has been extensively exploited [135]. When released into circulation, NPs are immediately exposed to high protein concentrations, thus determining the formation of a protein layer on their surface, altering their identity, and producing their so-called ‘biological identity’. Conversely, the surface of BEVs is often decorated by complex glycans that reduce the adsorption of proteins, thus maintaining the same composition. The BEVs are hence considered attractive for use as drug nanocarriers due to their high biocompatibility and ability to enter cells [136]. BEVs can also be considered a therapeutic platform due to their capacity to load and deliver active molecules [137].

## 6. Conclusions and Future Perspectives

The study of BEVs offers valuable insights into microbial ecology, biogeochemistry, and biotechnology. Further research is needed to unravel the mechanisms underlying EV production, decipher the functional roles of vesicles in marine ecosystems, and explore their potential applications in biotechnology. Understanding the intricate interplay between marine microorganisms, their vesicles, and the surrounding environment holds promise for advancing our knowledge of microbial life and harnessing its potential for the benefit of society and the environment. Their natural propensity to serve as vehicles for delivering bioactive compounds [138], combined with the recent advances in synthetic biology for engineering vesicles with tailored cargos, makes these natural nanoparticles a promising strategy for specific biotechnological purposes, opening new avenues for bioprospecting and innovation [139].

## Figures and Tables

**Table 1:** This table summarizes the vesicle sizes, types, and functions of various bacteria and cyanobacteria species.

Vesicle Size	Microorganisms	Vesicle Type	Functions	References
30–100 nm	*Prochlorococcus*, *Nostoc*, *Synechocystis*, *Gloeocapsa*, *Trichodesmium*	Small Vesicles (Exosomes)	Intercellular communication, carrying proteins, lipids, nucleic acids	[13,36,38,39]
100–300 nm	*Synechococcus*, *Anabaena*, *Microcystis*, *Spirulina*, *Oscillatoria*	Medium-sized Vesicles (Microvesicles/Ectosomes)	Nutrient acquisition, environmental signaling	[9,29,31,40]
300–700 nm	*V. cholerae*, *P. aeruginosa*, *B. subtilis*, *S. aureus*, *M. xanthus*, *Shewanella*, *E. coli*, *Rhodobacter sphaeroides*, *A. baumannii*, *H. pylori*	Large Vesicles	Cellular waste removal, containing fragmented DNA, extracellular polymeric substances, biofilm formation, environmental adaptation	[4,5,9,36,38,40,41]

**Table 2:** Biotechnological applications of BEVs.

Field	Function	
Drug Delivery	**Targeted delivery** to specific cells or tissues, enhancing the efficacy and reducing the side effects of treatments. **Controlled release** of therapeutic agents, improving the management of chronic diseases.	[127,130]
Vaccine Development	**As adjuvants,** to boost the immune response in vaccines. **As antigen presentation** to the immune system, thus enhancing the host’s response to pathogens.	[31,131]
Diagnostics	**Biomarkers** for the early detection of diseases. **Biosensors** for detecting environmental toxins or pathogens.	[2,24]
Bioremediation	**Pollutant degradation** through the incapsulation of enzymes. **Heavy Metal Removal:** They can also be engineered to bind and remove heavy metals from water and soil.	[132,133]
Nutraceuticals and Functional Foods	**Bioactive Compounds:** Vesicles can be used to deliver bioactive compounds in functional foods, enhancing their health benefits. **Probiotics:** They can encapsulate probiotics, improving their stability and efficacy.	[134]
Cosmetics	**Anti-aging:** Vesicles can deliver anti-aging compounds more effectively to the skin. **Skin Repair:** They can also carry compounds that promote skin repair and regeneration.	[130]
Agriculture	**Pesticide Delivery:** Vesicles can provide a controlled release of pesticides, reducing the amount of chemicals needed. **Plant Growth:** They can deliver nutrients and growth factors to plants more efficiently.	[31,130]
Nanotechnology	**Nanoreactors:** Vesicles can serve as nanoreactors for chemical reactions, providing a controlled environment at the nanoscale. **Nanocarriers:** They can be used as carriers for nanoparticles, enhancing the delivery of various materials.	[24,127]
